# Evaluation of the impact of childhood 13-valent pneumococcal conjugate vaccine introduction on adult pneumonia in Ulaanbaatar, Mongolia: study protocol for an observational study

**DOI:** 10.1186/s12889-021-11776-8

**Published:** 2021-09-23

**Authors:** Claire von Mollendorf, Mukhchuluun Ulziibayar, Bradford D. Gessner, Lien Anh Ha Do, Cattram D. Nguyen, Rohini Beavon, Bujinlkham Suuri, Dashtseren Luvsantseren, Dorj Narangerel, Adam Jenney, Eileen M. Dunne, Catherine Satzke, Badarchiin Darmaa, Tuya Mungun, E. Kim Mulholland

**Affiliations:** 1 Infection and Immunity, New Vaccines Research Group, Murdoch Children’s Research Institute, Royal Children’s Hospital, 50 Flemington Road, Parkville, VIC 3052 Australia; 2 Department of Paediatrics, The University of Melbourne, Parkville, Australia; 3 National Center for Communicable Diseases (NCCD), Ministry of Health, Ulaanbaatar, Mongolia; 4 Pfizer Vaccines, Collegeville, PA USA; 5 Pfizer Vaccines, Tadworth, Surrey UK; 6 Ministry of Health, Ulaanbaatar, Mongolia; 7 Department of Infectious Diseases, The Alfred Hospital, Monash University, Melbourne, Australia; 8 Department of Microbiology and Immunology, The University of Melbourne at the Peter Doherty Institute for Infection and Immunity, Melbourne, Australia; 9 Department of Infectious Disease Epidemiology, London School of Hygiene and Tropical Medicine, London, UK

**Keywords:** *Streptococcus pneumoniae*, Pneumococcal conjugate vaccine, Mongolia, Adult pneumonia, Surveillance, Indirect PCV impact, Nasopharyngeal carriage

## Abstract

**Background:**

Community-acquired pneumonia is an important cause of morbidity and mortality in adults. Approximately one-third of pneumonia cases can be attributed to the pneumococcus. Pneumococcal conjugate vaccines (PCVs) protect against colonisation with vaccine-type serotypes. The resulting decrease in transmission of vaccine serotypes leads to large indirect effects. There are limited data from developing countries demonstrating the impact of childhood PCV immunisation on adult pneumonia. There are also insufficient data available on the burden and severity of all-cause pneumonia and respiratory syncytial virus (RSV) in adults from low resource countries. There is currently no recommendation for adult pneumococcal vaccination with either pneumococcal polysaccharide vaccine or PCVs in Mongolia. We describe the protocol developed to evaluate the association between childhood 13-valent PCV (PCV13) vaccination and trends in adult pneumonia.

**Methods:**

PCV13 was introduced into the routine childhood immunisation schedule in Mongolia in a phased manner from 2016. In March 2019 we initiated active hospital-based surveillance for adult pneumonia, with the primary objective of evaluating trends in severe hospitalised clinical pneumonia incidence in adults 18 years and older in four districts of Ulaanbaatar. Secondary objectives include measuring the association between PCV13 introduction and trends in all clinically-defined pneumonia, radiologically-confirmed pneumonia, nasopharyngeal carriage of *S. pneumoniae* and pneumonia associated with RSV or influenza. Clinical questionnaires, nasopharyngeal swabs, urine samples and chest radiographs were collected from enrolled patients. Retrospective administrative and clinical data were collected for all respiratory disease-related admissions from January 2015 to February 2019.

**Discussion:**

Establishing a robust adult surveillance system may be an important component of monitoring the indirect impact of PCVs within a country. Monitoring indirect impact of childhood PCV13 vaccination on adult pneumonia provides additional data on the full public health impact of the vaccine, which has implications for vaccine efficiency and cost-effectiveness. Adult surveillance in Mongolia will contribute to the limited evidence available on the burden of pneumococcal pneumonia among adults in low- and middle-income countries, particularly in the Asia-Pacific region. In addition, it is one of the few examples of implementing prospective, population-based pneumonia surveillance to evaluate the indirect impact of PCVs in a resource-limited setting.

## Background

Adult community-acquired pneumonia (CAP) is an important cause of morbidity and mortality, especially in high-risk groups, both in high- and low-income countries. The burden of pneumococcal pneumonia among adults is not well characterised. Historical estimates were generally based on blood culture results only and therefore underestimated true disease burden [1], or included sputum which may reflect carriage and therefore overestimated burden [2]. Two luminex platform-based multiplex serotype-specific urinary antigen detection (SSUAD) assays have been developed and validated for identification of key *S. pneumoniae* serotypes in urine samples from adults with radiologically-confirmed CAP, which may assist in defining the burden of non-bacteraemic pneumonia [3, 4]. A meta-analysis that included studies utilising additional diagnostics such as the BinaxNOW® *Streptococcus pneumoniae* urine antigen test, attributed 27% of adult CAP to the pneumococcus [1]. This analysis, however, mainly included studies from high-income countries.

Nasopharyngeal pneumococcal carriage is a precursor to pneumococcal disease [5]. Pneumococcal conjugate vaccines (PCVs) protect against colonisation acquisition or density with vaccine-type (VT) and potentially some cross-reactive serotypes, while overall pneumococcal carriage prevalence is only slightly decreased (or unchanged) due to serotype replacement [6]. Nevertheless, reductions in transmission of VT serotypes have resulted in large indirect effects in high-income settings [7] because VTs tend to be more likely to cause disease than replacement serotypes. Indirect or “herd” effects of vaccines refer to the indirect protection of the non-vaccinated population with a reduction in disease or carriage within this group [8].

There are limited systematic data from developing countries demonstrating the indirect impact of childhood pneumococcal vaccination on adult pneumonia and results from developed countries cannot necessarily be extrapolated to developing countries [9]. For example, there is more frequent pneumococcal carriage in low- and middle-income countries with horizontal transmission between children and adults; developing country PCV schedules often do not include a post infancy booster dose (which may be critical to reductions in transmission) [10]; crowding and other factors may influence carriage density [11, 12]; indoor and outdoor pollution may damage upper airway mucosa [13] which may also then influence pneumococcal carriage; and transmission pathways may differ by geography such as the potential for serotype 1 transmission outside of early childhood in the African meningitis belt [14]. One of the few studies in a resource poor setting [15] – from Kenya – reported a 47 to 94% reduction in adult pneumococcal pneumonia incidence following the introduction of the 10-valent PCV using a 3 + 0 schedule (6,10 and 14 weeks) in the childhood immunisation programme. Even though other improvements in healthcare may have contributed to the decline, the authors argued that the indirect PCV effect could have a considerable effect on adult disease in a high burden setting [15]. While encouraging, these data need to be replicated in other settings. Because of the high burden of pneumococcal pneumonia outside of childhood, particularly among older adults and those with underlying cardiac and respiratory diseases, results will have importance for immunisation policies, including use of infant booster doses and the vaccination of older and other high-risk adults.

Viruses and pneumococci in the upper airway may interact to lead to a viral, pneumococcal or combined lower respiratory infection [16]. For example, many deaths during the 1918 influenza pandemic were attributed to secondary pneumococcal pneumonias [17]. In children, studies corroborate an association between respiratory syncytial virus (RSV) and pneumococcus [18] with declines reported in RSV- and influenza-associated pneumonia hospitalisation rates following PCV introduction [19]. Viral and viral-bacterial co-infections are not uncommon in adults with pneumonia [20, 21]. In adults with pneumonia who tested RSV-positive, those who had a bacterial coinfection, were more likely to have a more severe outcome (longer length of hospitalisation and more frequent ICU admission) than those who had no coinfection [22].

In Mongolia in 2015, respiratory disease was amongst the five leading causes of hospitalisations in all age groups (345.6 per 10,000 population per year), with 44% of these admissions due to pneumonia. Respiratory disease was also amongst the top five causes of mortality (2.0 per 10,000 population) in the same year [23]. There is strong seasonal variation in pneumonia incidence [24], with the peak season occurring during the winter months (approximately October until April), exacerbated by extreme winter temperatures with high domestic coal use and resultant air pollution [25]. Published data from Mongolia document influenza burden [24] but data on the burden and severity of RSV in adults is more limited in the region [26].

The Mongolian Government introduced 13-valent PCV (PCV13) using a 2 + 1 infant schedule (age 2, 4 and 9 months) in a phased approach over 3 years from June 2016 starting with the capital city of Ulaanbaatar and ending with introduction into the provinces from April 2019. Enhanced hospital surveillance for paediatric pneumonia was initiated in four district hospitals and one tertiary hospital from April 2015 to monitor the direct impact of PCV13 introduction [27, 28]. Community carriage surveys in infants and toddlers showed a reduction in PCV13 serotypes 1 year post-introduction [29]. There are currently no data on the prevalent pneumococcal serotypes in adults in Mongolia.

The main aim of the current study is to evaluate the indirect impact of childhood PCV13 vaccination on adult pneumonia by establishing adult hospital surveillance in the same districts as the paediatric programme. This study will also allow us to determine the incidence of all cause pneumonia in Mongolia in adults 18 years and older, and to determine the changes in pneumococcal serotypes causing adult disease over time.

## Methods

### Study objectives

The primary study objective is to evaluate the association between childhood PCV13 introduction and the trends in incidence of severe clinical pneumonia (see case definition) in adults 18 years and older in four districts of Ulaanbaatar over a 7 year period (Jan 2015 – Mar 2022).

The study has multiple secondary objectives:

1. To evaluate the association between childhood PCV13 introduction and the trends in incidence of (a) clinical pneumonia or (b) hypoxic pneumonia only (see case definition) in adults 18 years and older in four districts of Ulaanbaatar over a 7 year period (Jan 2015 – Mar 2022).
2. To evaluate the association between childhood PCV13 vaccination (during the early [2019–2020] and late [2021–2022] PCV13 introduction period) and the trends in adult pneumonia (see case definition) in four districts of Ulaanbaatar using the following endpoints: (a) all radiologically confirmed pneumonia, (b) definite pneumococcal pneumonia and (c) VT-pneumonia with a chest radiograph (CXR) consistent with CAP based on clinical assessment at time of hospitalisation.
3. To evaluate the association between childhood PCV13 introduction (during the early and late PCV13 introduction period) and the trends in the detection and density of pneumococci in the nasopharynx of adults admitted with pneumonia.
4. To assess changes in the detection of pneumococcal serotypes (VT and non-vaccine type [NVT]) associated with adult pneumonia in an era of ongoing childhood PCV vaccination.
5. Among adults hospitalised for pneumonia, who have children of vaccine eligible age living in the household, to compare adult cases and controls with respect to having a child in the house who received one or more doses of PCV13 compared with households having all PCV13-unvaccinated children. The case-control comparison will be done for three subgroups: VT (case) compared with NVT pneumococcal pneumonia (control); end-point radiologically-confirmed pneumonia (case) compared with other radiologic findings (control); hypoxic (case) compared with non-hypoxic (control) pneumonia.
6. To evaluate the association between PCV13 introduction in children on the trends in incidence and severity of adult pneumonia associated with viral pathogens (RSV or influenza) in four districts of Ulaanbaatar, during the early and late PCV13 introduction period, for the following endpoints: incidence of severe clinical pneumonia, clinical pneumonia, and radiologically confirmed pneumonia associated with RSV or influenza.

### Study setting

Mongolia has a well-structured health system with a clear referral pathway from primary family healthcare centres to secondary district hospitals, and finally tertiary hospitals for severe or complicated cases [30]. Most adults access public health care and most pneumonia cases are captured by the public health services in district and tertiary care facilities [23, 30, 31]. Hospital care is accessible with primary and secondary facilities in each district and payment is based on income. Private care and self-referral incur significant cost for individual patients.

Active hospital-based surveillance for adult pneumonia was established in 2019 in four district hospitals in four districts of Ulaanbaatar (Songinokhairkhan, Sükhbaatar, Bayanzürkh and Chingeltei). These are the same four districts with ongoing enhanced paediatric pneumonia surveillance. These four districts are the largest districts of Ulaanbaatar containing 70% of the population of Ulaanbaatar with a mixture of areas with formal and traditional informal (ger) housing. The vast majority of adult pneumonia cases in the four districts under study are expected to be captured by the surveillance programme [23, 30, 31]. Annual district-specific denominator data are available from the Mongolian National Statistics Office, allowing us to calculate incidence rates for our four study districts.

As part of the routine childhood immunisation schedule, two study districts had PCV13 introduced in June 2016 (Songinokhairkhan, Sükhbaatar), one district in July 2017 (Bayanzürkh) and the fourth district in March 2018 (Chingeltei). Catch-up campaigns, for children 12–23 months, with two PCV doses were instituted in 2016 and 2017, but not in 2018 [27]. There is currently no licensure or recommendations for adult pneumococcal vaccination in Mongolia, and the pneumococcal polysaccharide vaccine is not available in-country.

### Surveillance case definitions

#### Clinical pneumonia

Clinical pneumonia will be defined based on previously used case definitions [32]:

A diagnosis of pneumonia on admission, plus a discharge diagnosis of pneumonia, plus two or more of the following clinical signs and symptoms, at least one of which is respiratory:

- subjective fever/chills or documented fever (≥38.0 °C)
- new cough or change in chronic cough
- new or increased sputum production
- dyspnoea or tachypnoea (i.e. difficult breathing or respiratory rate > 25 breaths/minute)
- lung findings on auscultation (rales, decreased breath sounds)
- leucocytosis (> 11.0 × 10^9^ cells/l) or leukopenia (< 4.0 × 10^9^ cells/l)
- SaO_2_ < 90% or respiratory distress
- pleuritic chest pain

#### Severe clinical pneumonia

Severe clinical pneumonia will be defined as clinical pneumonia with two or more severity signs: presence of confusion (Glasgow coma scale < 15), respiratory rate ≥ 30 breaths per minute, hypotension (systolic blood pressure ≤ 90 mmHg or diastolic blood pressure ≤ 60 mmHg) or hypoxaemia (oxygen saturation < 90%) [33]. All pneumonia patients who are admitted in the ICU, receive pressor support in ICU or who die will be considered to have severe disease [34, 35].

#### Hypoxic pneumonia

Hypoxic pneumonia will be defined as clinical pneumonia with an oxygen saturation of < 90% in room air on admission as measured by pulse oximeter.

#### Radiological pneumonia

An adapted version of the WHO definitions for radiological pneumonia in children [36], which accounts for underlying chronic lung disease and significant infiltrates, will be used to diagnose CXR-confirmed disease in adults. Currently, Pfizer is using data from a randomised controlled trial of PCV13 among adults aged ≥65 years in The Netherlands to assess whether the WHO criteria for end-point pneumonia in children are valid for predicting pneumococcal pneumonia in adults. Similar to the definitions used in previous adult pneumonia studies [32], the following three endpoints are proposed:

- *Primary end-point pneumonia* will be defined as the presence of focal end-point consolidation (dense/fluffy opacity occupying portion/whole lobe or entire lung, that may or may not contain air-bronchograms) or pleural effusion in the lateral pleural space spatially associated with a pulmonary parenchymal infiltrate or if the effusion obliterated enough of the hemithorax to obscure an opacity.
- *Other infiltrates* will be defined as other patchy interstitial infiltrates without a pleural effusion or focal end-point consolidation. This will be a separate category as it may be difficult to confirm as acute lung disease in patients with a history of underlying chronic lung disease.
- *CXR-negative* will be defined as patients with no consolidation, infiltrate or effusion.

#### Definite pneumococcal pneumonia

Definite pneumococcal pneumonia will be defined as any clinical pneumonia admission with a positive blood or pleural fluid culture or a positive pneumococcal urine test from BinaxNOW® urinary antigen test; or any radiographically confirmed pneumonia (primary end-point pneumonia or any infiltrate consistent with CAP) with a positive multiplex SSUAD diagnostic assay result.

#### Viral pneumonia co-infections

Pneumococcal viral co-infection will be defined as any clinical or radiological pneumonia case where both *S. pneumoniae* and RSV or influenza are detected in nasopharyngeal samples. *S. pneumoniae* will be identified by quantitative real-time polymerase chain reaction (PCR) targeting the *lytA* gene (qPCR) [37] and microarray as per testing algorithm [29]. RSV or influenza will be identified by RT-PCR [27]. In addition, for radiologically-confirmed pneumonia cases, pneumococcal viral co-infection will include cases with a positive pneumococcal urinary antigen test and RSV or influenza detected on nasopharyngeal sampling [27].

### Study design

The surveillance programme includes both retrospective (Jan 2015 - Feb 2019) and prospective (March 2019–March 2022) data collection. The study has a number of different components, including prospective pneumonia hospital-based surveillance with a nested case-control study, retrospective pneumonia chart review, data collection regarding other clinically relevant interventions and trends in cardiovascular and renal disease admissions as “control” conditions, and enrolment of controls without evidence of respiratory disease for validation of the urine antigen test [4].

#### Prospective adult pneumonia hospital-based surveillance

##### Recruitment procedures

Prospective adult pneumonia hospital-based surveillance started on 1 March 2019. Participants are adults 18 years and older who are admitted with pneumonia to one of the four participating district hospitals and enrolled in the surveillance system. Hospital staff actively identify and screen all new adult respiratory admissions daily (from admission and other wards) to assess suitability for the study. Details regarding all patients screened, their eligibility and whether they were enrolled or not, as well as reasons for exclusion are captured on a screening/ enrolment log. Study staff review all admissions on a weekly basis to ensure that no eligible patients were missed. Any missed patients will be enrolled at time of identification.

If patients meet the clinical pneumonia case definition and provide written consent to be included in the study, clinical information is collected using an adapted case report form with details regarding presentation (e.g. oxygen saturation on admission), clinical management (e.g. provision and duration of supplementary oxygen) and risk factors for carriage and disease. If patients are unable to give consent – e.g., because of mechanical ventilation, severe illness, or mental incapacity – permission is obtained for enrolment from the next of kin to prevent severe cases from been missed.

Recorded risk factors include previous admissions for pneumonia and other conditions, level of education, recent migration to Ulaanbaatar, household size, number of children in the household, PCV vaccination status of children in the household, dwelling type, history of working in a mine, tobacco smoking, alcohol use, type of heating (coal, briquettes, electric or central) and type of stove in the household, history of tuberculosis, self-reported and documented comorbidities and pregnancy. Details regarding underlying medical conditions, antibiotic use and severity markers, e.g. blood pressure, respiratory rate, and level of consciousness according to the AVPU (“alert, verbal, pain, unresponsive”) scale and Glasgow Coma Scale [34, 35] are collected during medical record review. Prospectively enrolled participants will have digital CXRs, nasopharyngeal swabs and urine collected.

##### Number of subjects

Based on a review of admission rates in previous years we anticipate enrolment of approximately 1000–1500 adults with pneumonia per year in the prospective surveillance system in the four districts. All adults meeting the pneumonia surveillance case definitions will be invited to participate in the study.

##### Eligibility, inclusion and exclusion criteria

Any adult 18 years of age or older admitted to one of the four participating district hospitals and meeting one of the surveillance case definitions for pneumonia will be eligible for inclusion. Exclusion criteria include not residing in one of the four districts for the last 3 months and readmission for pneumonia within 14 days of a previous admission for pneumonia.

##### Informed consent

Only adults who consent will be enrolled. If the nurse or doctor is unable to obtain consent from a patient who is severely ill, ventilated, or otherwise incapacitated, the nurse or doctor will contact the patient’s next of kin to obtain permission to enrol the participant in the study. The next of kin will be provided with the same information as for a participant and be asked to sign the consent form on behalf of the participant.

#### Nested case-control study

This substudy will only include adults residing in households with children eligible to receive PCV13. Vaccine eligibility will be based on the relevant district vaccine introduction timelines. We will confirm household level vaccination data, obtained by verbal report, using clinic or electronic immunisation records if available. Children eligible to receive PCV13, who are identified as unvaccinated, will be referred to their local health centre for catch-up according to local guidelines.

When we identify an adult, who is hospitalised for pneumonia and who has children of vaccine eligible age living in the household, we will seek to enrol the adult and their household in the case-control study. We then conduct three separate case-control analyses (defined below) with respect to having any child in the house who received one or more doses of PCV13 compared with households having all PCV13-unvaccinated children.

The case-control comparison will be done for three endpoint-based subgroups.

- Among adults hospitalised with pneumonia and with a CXR consistent with CAP based on clinical assessment at time of hospitalisation, VT pneumococcal pneumonia (case) compared with NVT pneumococcal pneumonia (control) based on serotype-specific urine antigen assays.
- Among adults hospitalised with pneumonia and with a CXR obtained, those with findings meeting adapted World Health Organization (WHO) paediatric radiological criteria (case) compared with those who have any other radiologic finding (control). This analysis is based on the assumption that pneumococcal pneumonia is more likely to have WHO endpoint consolidation CXR findings than non-pneumococcal pneumonia.
- Among adults hospitalised with pneumonia and with pulse oximetry performed, those with hypoxia (case) compared with those without hypoxia (control). This analysis is based on the assumption that pneumococcal pneumonia is more likely to be hypoxic than non-pneumococcal pneumonia.

These analyses will be controlled for relevant confounders, such as age and housing type.

#### Retrospective pneumonia chart review

##### Chart review procedures

To provide additional baseline data on adult pneumonia admissions in the four districts, including inter-district variability in the incidence of clinical pneumonia prior to PCV13 introduction, we undertook a retrospective chart review of all adult pneumonia admissions to the adult hospitals in these districts from January 2015 to the start of prospective adult hospital-based surveillance in March 2019. Data extraction was undertaken by trained staff and included clinical data, demographic data, district and subdistrict (khoro) of residence, age, date of birth, length of hospital stay, outcome and severity markers. No urine, nasopharyngeal swabs or digital CXRs were available for this period.

Staff reviewed admission registers for potential pneumonia admissions and then reviewed the relevant medical records to confirm whether patients met predefined pneumonia case definitions. Overall respiratory admission numbers and pneumonia admission numbers were also collected. To ensure that no cases were missed during the retrospective review period, staff retrieved all medical records for review where any respiratory system disease diagnosis was recorded in the admission log records to confirm whether the patient had pneumonia which met the study case definition or not. In addition, all intensive care unit (ICU) admissions and deaths were reviewed to ensure that severe disease cases were not missed. The medical records were analysed for the same severity markers as those recorded prospectively. The definition of severe clinical pneumonia remained standard across the pre- and post-PCV period.

##### Number of subjects

There were 2500–3200 annual adult admissions at all four district hospitals from 2015 to 2018 identified from admission books, with 1200–1600 total pneumonia cases per year who had medical records extracted. Between 850 and 1220 adult pneumonia cases per year met the study eligibility criteria and were enrolled retrospectively. An additional 400 cases were enrolled in January and February 2019.

##### Eligibility, inclusion and exclusion criteria

Medical records were reviewed for all adults admitted to one of the four participating district hospitals with pneumonia from January 2015 to the start of the prospective surveillance in March 2019. Those adults who met one of the surveillance case definitions and lived in one of the four participating districts were included.

#### Clinical information collected on other relevant interventions and control condition

Ongoing information is collected on any changes in clinical practice or policies affecting pneumonia admissions instituted over the study period. This includes changes in antibiotic policies, antibiotic prescription practices, annual influenza vaccination campaigns (number of vaccine doses dispensed and coverage by age group) and changes in admission criteria.

There was a large nationwide measles outbreak in Mongolia which began in March 2015 and had two waves, March to September 2015 [38] and October 2015 to June 2016 [39]. A total of 49,908 suspected cases were reported, and 33,947 cases were confirmed. During the first wave, 9671/14,010 (69%) cases occurred among adults ≥15 years and 11,346/19,937 (57%) in the second wave [39]. We will also need to account for the potential changes in hospital admissions due to the ongoing COVID-19 pandemic and related public health interventions from 2020. To account for changes in hospital admissions due to the ongoing COVID-19 pandemic, we are collecting data on lockdown and other public health restrictions, COVID vaccination rates and vaccination status for admitted pneumonia cases. In addition, all adult respiratory cases are tested for SARS-CoV-2 on admission to hospital using rapid antigen testing and confirmation by PCR. Only SARS-CoV-2 negative pneumonia cases are enrolled into the surveillance programme.

To ascertain whether health-seeking behaviour and admission practices may have changed between the retrospective and prospective periods, admissions for two “control” conditions, cardiovascular and renal disease, will be collected over the entire study period (2015–2022) at all four hospitals for all adult patients. Weekly (prospective) or monthly (retrospective) numbers will be collected by age group and diagnosis category (for cardiovascular disease: ischaemic heart disease, heart failure, hypertension, cardiomyopathy, heart valve disease, vasculitis and other cardiovascular disease; for renal disease: pyelonephritis, renal failure, cystitis, glomerulonephritis and other renal disease).

#### Control enrolment for serotype-specific urinary antigen detection (SSUAD) testing

##### Recruitment

To establish positivity cut-off limits for the SSUAD assay in the Mongolian population (see “urine processing” section for details), urine samples were collected from 400 adult controls in June and July 2019 (i.e. during summer months, to reduce the probability of enrolling patients with recent or current respiratory infections). Staff screened all new non-respiratory adult admissions in the medical and surgical wards on a daily basis to assess suitability as a control for the study. If patients met the control inclusion and exclusion criteria and they provided written consent to be included in the study, clinical information was collected by the doctor using a questionnaire adapted from the case questionnaire. The control questionnaire included basic demographics, medical history and medication information. Controls were required to sign an informed consent form before a urine sample was collected. Any controls that tested positive on BinaxNOW® urinary antigen test, were disenrolled as controls. None of the seven controls who tested positive reported a recent admission for pneumonia [40].

Details regarding all patients screened, their eligibility and whether they were enrolled or not, as well as reasons for exclusion were captured on a screening/enrolment log. An equal number of controls were enrolled from each of the four hospitals and based on determined targets over four age groups (18–25, 26–45, 46–65 and > 65 years).

##### SSUAD controls inclusion criteria

Controls had to meet the following inclusion criteria to be eligible for study enrolment:

- Able to provide written informed consent and understand all relevant study components.
- Aged 18 years or older.
- Willing and able to provide a urine sample.

##### SSUAD controls exclusion criteria

Controls presenting with any of the following criteria were not included in the study:

- Possible pneumonia or another respiratory infectious disease or signs of pneumococcal disease, with evidence of active infection.
- Residing in a long-term care facility.
- Known bronchial obstruction or a history of post-obstructive pneumonia. Chronic obstructive pulmonary disease was allowed, provided there had not been an exacerbation within the 3 months prior to enrolment.
- Primary lung cancer or another malignancy metastatic to the lungs.
- Fever (temperature of ≥38.0 °C measured by a healthcare provider).
- Significant immunosuppressive disease such as AIDS, leukaemia, etc.
- Received PCV and/or pneumococcal polysaccharide vaccine (PPSV) within the past 30 days.

#### Clinical and laboratory methodology for prospective study

##### Nasopharyngeal swab collection and testing

Nasopharyngeal swabs are collected and stored according to WHO guidelines [41]. Swabs are collected by designated trained nurses at each hospital and placed into a vial containing STGG medium and stored in a refrigerator in the specimen collection area at each hospital. Utilising the same transport and processing procedures as the paediatric surveillance project [27] swabs are transported to the National Center for Communicable Diseases (NCCD) microbiology laboratory, where they are vortexed and aliquoted. Samples shipped to the Murdoch Children’s Research Institute (MCRI) are screened for pneumococci using qPCR [37]. Samples that contain pneumococci undergo molecular serotyping by microarray following culture-amplification [42]. Testing for RSV and influenza will be conducted at the NCCD Virology laboratory or MCRI, using a validated multiplex real-time RT-PCR which has been validated in other studies in Vietnam [43] and in the paediatric surveillance project in Mongolia [27].

##### Urine processing

Urine samples from adult cases enrolled prospectively in the adult surveillance programme, will be tested at the NCCD bacteriology laboratory in Mongolia using a rapid pneumococcal urinary antigen test (BinaxNOW®, Alere North America, Orlando, Florida). The BinaxNOW® *Streptococcus pneumoniae* test is an immunochromatographic membrane assay test used to detect pneumococcal soluble antigen in human urine. It detects the pneumococcal C-polysaccharide which is found in the cell wall and is common to all serotypes. BinaxNOW® urine testing conducted at the NCCD bacteriology laboratory will be conducted by two designated trained laboratory technicians according to study standard operating procedures to ensure consistency of results.

Additional urine will be frozen in ultra-low temperature (− 70 °C) freezers before being shipped to the Pfizer Laboratories, Pearl River, USA for pneumococcal testing using multiplex SSUAD assays that assesses the 13 serotypes in PCV13 (UAD1) [4] and the additional 11 serotypes in 23-valent pneumococcal polysaccharide vaccine (PPSV23) (UAD2) [3]. Positivity cut-off limits for the SSUAD assay will be established based on antigen concentrations read off a standard curve for each serotype using the 400 urine specimens collected from control subjects as described above. While UAD cut-points have only rarely been reset based on evaluation of local healthy control subjects, this step ensures test validity and high specificity.

##### Processing of other samples and CXRs

Any pleural fluid collected will be cultured and blood cultures will be taken according to standard clinical practice. Blood culturing practices may differ between the four hospitals and will not be done for all adult pneumonia cases. Pleural fluid will be tested by *lytA* qPCR in Mongolia if possible, otherwise shipped and tested at MCRI.

CXRs will be performed and archived for all pneumonia cases. CXRs will be digitalised and uploaded in an electronic database for re-reading according to adapted standardised WHO criteria. CXRs will be reread by two independent radiologists who are trained in WHO methodology, and results will be entered into the electronic database. Any disagreement in results between the two readers will be resolved by a third radiologist.

#### Statistical methods

##### Sample size estimation

Power calculations were based on a generalised linear mixed model to account for the staggered vaccine introduction and clustering within the four study districts [44]. Calculations assumed that for clinical pneumonia the data from 2015 to 2022 would be included. Calculations assumed an average of 13,271 person-years in each district per monthly interval and an intra-cluster (i.e. intra-district) correlation of 0.2. Considering the timing of vaccine introduction and a significance level of 0.05, the study has 92% power to detect a 20% decrease in severe pneumonia incidence rate from 9.8 to 7.8 per 10,000 person-years. Calculations were performed in Stata version 15 using the “stepped wedge” command [45].

##### Statistical analysis plan

**Fig. 1:**
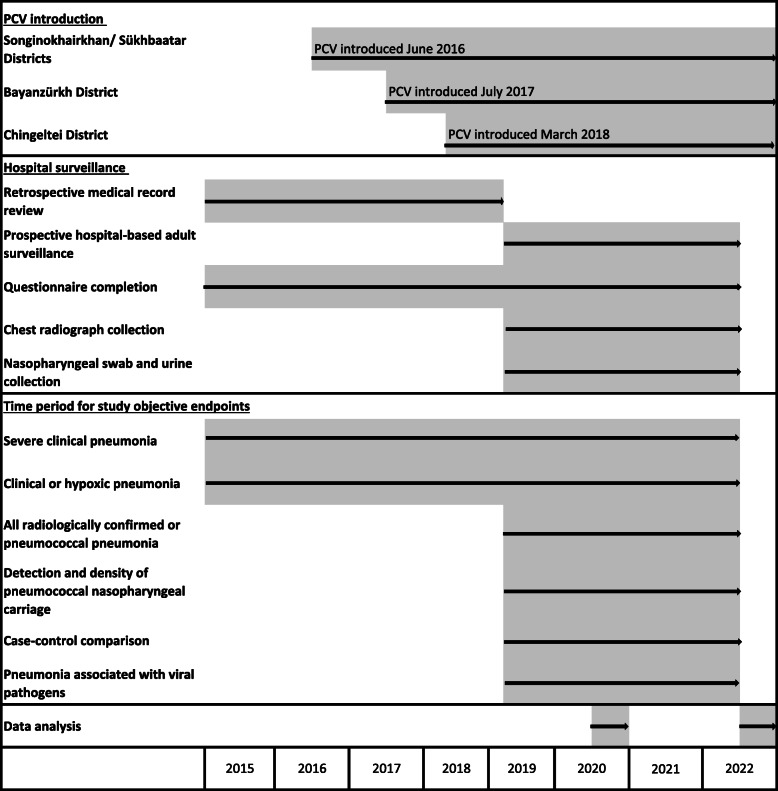
Schematic diagram of study design and timelines

The primary analysis endpoint will be the change in severe clinical pneumonia incidence rate ratios (IRR) comparing the pre- and post-vaccine periods over 87 months in all four districts (Songinokhairkhan and Sükhbaatar with 17 months pre-PCV and 70 months post-PCV; Bayanzürkh with 30 months pre-PCV and 57 months post-PCV; and Chingeltei with 38 months pre-PCV and 49 months post-PCV). Retrospective data from 2015 will be used to contribute to pre-vaccine case numbers for the primary outcome. See Fig. 1 showing the schematic study timelines.

The outcome variable for this analysis will be monthly district-level counts of severe clinical pneumonia. The IRR will be estimated using a mixed effects Poisson loglinear regression model (or a negative binomial model if there is over-dispersion) fitted to monthly counts, with log-transformed population denominators included as an offset. The model will include a fixed effect for PCV13 (indicator variable for PCV13 introduction which will depend on the district as PCV13 introduction was staggered), a fixed effect for time (time since study commencement in months) to account for temporal trends, and a random effect for district. The PCV13 term will be exponentiated to obtain the incidence rate ratio for the vaccine effect. This model assumes that there is uniform vaccine impact across the districts. Sensitivity analyses will explore whether the vaccine impact differs across districts and will include seasonality and employ changes in vaccine coverage as opposed to fixed PCV13 effects. A subgroup analysis will explore rate changes in different age groups (18–25, 26–45, 46–65 and > 65 years). We will also do a sensitivity analysis with a 1 year “lag period” for indirect PCV effect (as we don’t expect the indirect effect to be instantaneous).

For the secondary pneumonia endpoints, no pre-PCV13 radiological and urine data are available. Consequently, we will explore trends over time in the post-PCV13 introduction period, incorporating district and time since PCV13 introduction.

We will conduct a nested case-control analysis (“test-negative design” [46]) to measure the indirect PCV13 vaccine effectiveness among adults living with children eligible to be vaccinated with PCV13. The model will account for district and time since vaccine introduction. We will compare the odds of receiving PCV13 among household children between cases and controls, defined as: 1) adults with vaccine type (cases) versus non-vaccine type pneumonia (controls), 2) among adults who had a CXR, WHO defined radiological confirmed pneumonia (cases) versus no radiological pneumonia (controls) and 3) among adults who had pulse oximetry, hypoxic (cases) versus non-hypoxic (controls) pneumonia.

Numbers of cases hospitalised with cardiovascular or renal disease in the four district hospitals will be collected from 2015 to 2022 as two separate “control” conditions to help determine whether changes over time are related to secular trends or vaccine effects. We will include the control condition in the model for severe clinical and clinical pneumonia as a covariate. We will have an “uncontrolled” model as the main analysis, and a “controlled” model as a sensitivity analysis.

Chi-square tests will be used to evaluate changes in PCV13 and non-PCV13 serotypes over time, comparing the proportions of both identified in year one to year three, for each age group.

An interim analysis will be conducted for the primary clinical outcome in December 2020 and will be conducted in the same way as the final analysis except for a shorter follow-up time (71 months). The final analysis, which will include all primary and secondary objectives as well as laboratory data (nasopharyngeal swabs and urine), will be conducted once data collection, data cleaning and laboratory analysis has been completed - planned end of 2022.

A separate detailed statistical analysis plan including details related to the secondary analyses, nested case-control study and changes to model assumptions considered for sensitivity analyses has been approved by the study statisticians.

## Discussion

We have designed an active hospital-based adult pneumonia surveillance programme to evaluate the indirect impact of childhood PCV13 vaccination on different adult pneumonia endpoints, determine the incidence of all cause pneumonia in Mongolia in adults 18 years and older, and determine the changes in pneumococcal serotypes causing adult disease over time. This study will collect over 4 years of retrospective pneumonia data (over 4500 cases) and 3 years of prospective pneumonia data (an estimated 3500 cases).

Childhood PCV13 programmes provide indirect protection to adults through reductions in carriage and transmission, in turn enhancing the vaccine’s public health value [47, 48]. Such a full public health accounting is particularly important for high burden lower-income countries that are graduating from Gavi, The Vaccine Alliance support. In addition, it is important to collect data evaluating potential limitations of paediatric PCV13 programs in protecting adults. Invasive pneumococcal disease surveillance data from the UK has demonstrated that some vaccine serotypes continue to circulate and cause disease, with increasing incidence, despite a robust paediatric immunisation program [49]. Similarly in adults with CAP in the UK, 12.2% of disease was due to the additional PCV13 serotypes (predominantly serotype 3) and 15.1% due the additional PPSV23 serotypes [50]. A prospective surveillance study in the United States which recruited adults from 21 acute care hospitals from October 2013 to September 2016, detected PCV13 serotypes in 4.6% of all patients and 4.2% of elderly patients ≥65 years with radiologically-confirmed pneumonia [51]. Over the study period a small decrease was seen in PCV13 serotypes in those groups recommended to receive PCV13 (≥65 years and those with high risk conditions), but not in the groups where PCV13 was not recommended routinely [51]. Other studies have demonstrated some reductions in all-cause or clinical pneumonia from direct PCV13 immunisation of older adults despite long-standing paediatric PCV programs [52, 53]. If such results are duplicated in Mongolia it will inform decision makers on whether PCVs, including planned 15 and 20-valent vaccines, will likely have a role in directly protecting adults.

Previous studies have shown increases over time in the indirect effect of childhood vaccination with PCV7 and PCV13 on adult invasive pneumococcal disease [54] and pneumonia [15]. Indirect impact is not only related to time since vaccine introduction, but it is also affected by other factors such as vaccine coverage and timing. The point at which indirect effects are observed in different settings may therefore be variable. Vaccine introduction was completed in the study districts in 2018 and we therefore assigned 2019/2020 as the early PCV introduction period for comparison to the last 2 years of the study 2021/2022.

### Study strengths

Documenting the impact of PCVs in Asian countries is limited by only a few sites having denominator-based surveillance for invasive disease or pneumonia and the ability to accurately investigate and document pneumonia cases. Our active hospital-based surveillance as well as population district level denominators will allow us to determine the incidence of adult pneumonia in Ulaanbaatar, Mongolia and the indirect impact of paediatric PCV13 use on adult outcomes. The sampling and laboratory methods used are consistent with other research studies that have described nasopharyngeal carriage in adults [55]. We also used gold-standard molecular methods to detect pneumococcus in the nasopharynx [42]. The use of the SSUAD assay, which detects PCV13 and PPSV23 VT pneumococcal polysaccharides in the urine of adults with suspected pneumonia, will allow us to determine the indirect effect of PCV13 on PCV13 VT non-bacteraemic pneumococcal CAP in adults.

PCV13 was introduced in a phased manner (at district level) in Mongolia. This will allow for between and within district comparisons and allow us to explore heterogeneity in effects between districts and possibly account for some unmeasured confounders.

Our study collects data on other factors which may influence trends in respiratory disease, as well as a control condition, and incorporates different study designs, which will help us to interpret the changes over time in adult pneumonia following PCV introduction.

### Study limitations

While our study includes four districts containing over 70% of Ulaanbaatar’s population, we did not include five of the city’s districts. Therefore, our findings may not be applicable to rural areas of the country. We only have 1 year of data pre-PCV13 introduction for all districts, however due to the phased introduction of the vaccine this period is extended in some districts. We have included both retrospective and prospective surveillance data which may potentially differ; however a standard clinical medical record was used in the district hospital sites over the entire period from 2015 with the same available clinical information for both periods, reducing the risk of discrepancies. We do not have pneumococcal carriage data for the retrospective period and have therefore chosen clinical endpoints as our primary study objective. The 2015/16 measles outbreak and annual variations in influenza and RSV cases may impact pneumonia outcomes; however, as we have administrative data available for both, we plan to incorporate this information into the descriptive interpretation of study results.

The adult surveillance programme in Mongolia will provide data to help guide paediatric and adult PCV immunisation policy in the country. It will contribute to the incomplete evidence available on the burden of pneumococcal pneumonia in adults in low resourced countries, particularly in the Asia-Pacific region. The study is one of the few active population-based pneumonia surveillance programmes evaluating the indirect impact of PCV in a resource-limited setting. With increasing introduction of PCV in low-resourced settings, the methods, experiences and lessons learned from our study may be used to guide the development of such systems in other countries.

## Data Availability

Not applicable – manuscript does not contain any data.

## Abbreviations

CAP: Community-acquired pneumonia
CXR: Chest radiograph
IPD: Invasive pneumococcal disease
ICU: Intensive care unit
IRR: Incidence rate ratio
NVT: Non-vaccine type
PCR: Polymerase chain reaction
PCV: Pneumococcal conjugate vaccine
PPSV: Pneumococcal polysaccharide vaccine
RNA: Ribonucleic acid
RSV: Respiratory syncytial virus
SSUAD: Serotype-specific multiplex urinary antigen detection assay
WHO: World Health Organization
VT: Vaccine type
